# Impact of the Physical Activity on Bullying

**DOI:** 10.3389/fpsyg.2019.01520

**Published:** 2019-07-02

**Authors:** Inmaculada Méndez, Cecilia Ruiz-Esteban, Enrique Ortega

**Affiliations:** ^1^Department of Developmental Psychology and Education, University of Murcia, Murcia, Spain; ^2^Department of Physical Activity and Sports, University of Murcia, Murcia, Spain

**Keywords:** bullying, secondary education, adolescence, physical activity, gender

## Abstract

Relationship problems among school children can lead to bullying situations. In this regard, it should be noted that, among healthy lifestyle habits, sports practice (non-competitive) promotes responsibility and improves coexistence. The objective of the present study was to analyze the incidence of the frequency of practice of healthy physical activity on the risks of students directly involved in school bullying (harasser and victim) by gender. The participants of the study were 1,248 students of Compulsory Secondary Education with ages between 11 and 18 (*M* = 14.42, *SD* = 1.43), being 50.8% males. The results of the study indicated that students who practiced physical activity in the recommended frequency rated as healthy, at least four or more times per week, had higher values in the indicators of aggressiveness than students who practiced with a lower frequency, appreciating a greater relationship between both variables in male rather than in female students. The study will make progress in preventive and intervention programs whose central axis is the promotion of physical activity and healthy sport (non-competitive) among students involved in situations of bullying. Likewise, teacher training in the recognition of bullying is considered a priority, providing them with guidelines for action.

## Introduction

Due to the fact that the American College of Sports Medicine (ACSM) together with the Centers for Disease Control and Prevention (CDC) established some recommendations on the types and amounts of physical activity needed to improve and maintain health (Haskell et al., 2007; World Health Organization [WHO], 2010), programs which promote physical activity in schools are essential (Medeiros et al., 2018). Therefore, organized sport must complete, but not replace, a regular physical activity, since the physical activity is a formative tool in the educational context (Guzmán-Guzmán, 2010). Specifically, in Spain, the data provided by the Delegación del Gobierno para el Plan Nacional sobre Drogas [DGPNSD] (2016a, b)Government Delegation for the National Plan on Drugs –show that 70.8% of the adolescents practice sport weekly, although this percentage decrease as the age increases. In particular, the Annual Directory of Sport Statistics (Subdirección General de Estadística y Estudios, 2018) highlights from the Survey on Sport Habits in Spain (Subdirección General de Promoción Deportiva y Deporte Paralímpico, 2015) that inactive students who do not carry out any type of physical-sporting activities outside school hours, represent a 9% of the school population. Thus, 53.5% of the population from 15 years old had practiced sport in the last year and 47% of the student population had carried out some physical-sporting activity during the break. Therefore, nowadays, there is a widespread concern for the low level of physical activity among teenagers, something that can be linked to high levels of overweight and obesity (Ramos et al., 2016).

Accordingly, the practice of physical activity (PA) in the adolescence plays an essential role in the promotion of healthy lifestyles (Campo-Ternera et al., 2017) as a lesser tendency toward drug use is shown (Hernando et al., 2013; Galán et al., 2014; González et al., 2016). Reciprocity between the self-concept of the physical condition and the competence perceived, as well as of the general health, physical function, mental health and vitality, has been found (Palomino-Devia et al., 2018). Thus, a greater physical activity increases the quality of life associated to health and self-esteem (Zurita-Ortega et al., 2018). Teenagers with a more self-determined profile perceive a greater teacher’s support toward autonomy, greater competence and social relationships, and greater satisfaction in the practice of physical education or even a greater practice of physical activity (Fin et al., 2017). Most adolescents practice team sports, which provides greater possibilities of enjoyment and, at the same time, promote social relationships (González et al., 2016). With reference to gender, some authors have evinced that the percentage of inactive women being higher than that of men (Chahín-Pinzón and Libia, 2011; Hernando et al., 2013; Hutchens et al., 2016; Medina et al., 2018) even at the lower socioeconomic levels (Moreno-Maldonado et al., 2016; Ramos et al., 2016; Chzhen et al., 2018). There are more men in federated sports (Castro-Sánchez et al., 2016). Women show a higher social status (popularity and respect) in the school center while men’s is higher in the Physical Education (PE) classes (Santos et al., 2018).

Likewise, some authors demonstrate that the practice of physical activity (PA) (non-competitive) is an excellent means for the transmission of values (Portolés and González, 2015) and helps to promote prosocial attitudes (González et al., 2016) so it can be helpful in the prevention and treatment of bullying and victimization and have a lower risk of developing aggressive and deviant behaviors (Pelegrín et al., 2010). Other studies make reference to the increase of aggressiveness produced by competitive sports (students replicate the violent situations, e.g., football and basketball to a greater extent) (Chacón-Cuberos et al., 2015; Subdirección General de Promoción Deportiva y Deporte Paralímpico, 2015). Thus, those teenagers who practiced sport on a regular basis showed a higher overt aggressiveness than sedentary teenagers, because started competing (Zurita-Ortega et al., 2015). Martínez-Baena and Faus-Bosca (2018) mention that there are scarce studies which relate bullying in the practice of physical activity. It has been proved that certain factors such as being overweight, having educational needs or deficient motor skills, etc., can be a risk factor for being bullied in the PE classes (Bejerot et al., 2011; Bejerot et al., 2013; Healy, 2014). Therefore, a moderate physical activity which is oriented toward disciplines such as football or athletics implies a greater victimization in all dimensions while one oriented toward the martial arts or popular games involves lower rates of victimization. Consequently, the amount of physical activity carried out and the type of sport practiced can act as regulators in the victimization for bullying. In relation to differences by gender, the following points are highlighted: significant differences in the dimension of indirect verbal violence in basketball, in violence for social exclusion in the martial arts, and in violence via new information and communication technologies in athletics (Medina Cascales and Reverte Prieto, 2019). Thus the report issued by the Fundación ANAR – Foundation for the Help of Children and Adolescents at Risk – (Ballesteros, 2018) shows that victims suffer a higher number of violent acts in comparison to previous reports (2.6%), observing that the situations of bullying are more and more violent, tougher and in more places. Then, bullying with medium or high seriousness implies up to 97% of cases. That is to say, bullying has become a problem on a global scale (Olweus, 2013). Furthermore, in Spain the studies evince that 9.3% of the students had been victims of bullying and 5.4% had been aggressors as it is pointed out in the report issued by Save the Children (Sastre, 2016) in the same way Sánchez-Queija et al. (2017) refer to an increase of victimization (20%). Moreover, both men and women are involved in the different forms of aggression (Chacón-Cuberos et al., 2015; Martínez-Martínez et al., 2017; López-Castedo et al., 2018; Medina Cascales and Reverte Prieto, 2019). Indeed, with reference to victimization, 10.6% of women had suffered bullying (Sastre, 2016).

Due to that there are scarce studies which relate bullying in the practice of PA, outside school, the aim of this study was to analyze the impact of the amount of healthy physical activity on the risks for the students directly involved in bullying (bully and victim) according to gender.

## Materials and Methods

### Participants

The participants in the study were 1.248 students of Compulsory Secondary Education from different geographical areas of the Region of Murcia in Spain (73% urban and 27% rural areas) with an age range from 11 to 18 (*M* = 14.42; *SD* = 1.43), being 50.8% female and 49.2% men; 83.7% were Spanish and 16.3%, foreigners. We consider this sample as representative (with a maximal error of 3%) of the Secondary pupils of the Region of Murcia. The inclusion criteria used were the following: students in compulsory secondary education with ages between 11 and 18 years who attended school the day of the test. Exclusion criteria were the following: absence the day of the questionnaire and substantial deficit in the mastery of the Spanish language.

### Design and Procedure

This research is a transversal descriptive study. The participants in this study were students selected from secondary schools in Murcia (Spain). After obtaining the permissions, students were approached at their own classrooms in school. Researchers explained the objectives of the study and the instruments that would be used. Participation was voluntary and confidential.

The study protocols were approved by the Ethics Committee of the University of Murcia, and the study was performed in accordance with the approved guidelines and the Declaration of Helsinki, with written informed consent from all participants. To participate in the study, informed consent of the parents was required. The protocol was approved by the Ethic Committee for Clinic Investigations of the University of Murcia.

One session of 50 min was used to complete the tests (20 min for the Bull-S Test, 20–25 min for the second scale).

### Instruments

To measure aggressiveness or victimization in bullying, the Bull-S test, Assessment Test of Aggressiveness (version 3.3) was used (Cerezo, 2012). It consisted of 15 direct choice Likert items and was addressed to all individuals in the group-class. The test had three dimensions: Dimension 1, Sociometric status (4 items by peer nominations); Dimension 2, Bullying dynamic (6 items by peer nominations), and Dimension 3, Situational perception (5 Likert scale items). In this study, we used dimension 2. It provided information on the students who stood out in at least 25% of each profile linked to bullying dynamics: the aggressor’s profile and the victim’s role. The features associated to the aggressor profile were related to continuous items: physical strength, aggressiveness and provoking behavior; and those associated with the victim role: cowardice, victimization and fixation. Individuals who scored significantly high in victimization and fixation were classified as provocative victim. Cronbach’s alpha coefficient was 0.68 for total scale scores (0.73 for aggressors and 0.84 for victims) (Cerezo, 2012). In this study, the coefficient was 0.68 for total scale scores (0.83 for aggressors and 0.84 for victims). Example of items: Who are the victims?

To measure physical activity and health, the scale applied was based on the “National Survey on Drug Consumption in Secondary School Students” (ESTUDES), issued by the Government Delegation for the National Plan on Drugs – DGPNSD – (2016) and APAL-Q (Zaragoza et al., 2012). From the ESTUDES test the items refer to the frequency of realization of sports habits taking into account the place of performance, the causes in case of not doing, the consideration of being in good physical shape and if you consider the individual that puts your health at risk. On the other hand, APAL-Q is a short self-report physical activity questionnaire. The scale contained five questions. The answers were coded on a 4-point Likert scale (1 is the lowest value and 4 the highest). Example of items: do you do physical-sporting activities outside school? Cronbach’s alpha coefficient was 0.76 for total scale scores. The test included socio-demographic variables too: gender (male/female), age, grade, origin (Spanish/foreigner), course repetition (yes/no), nature of the school (public/private/semi-private) and geographical location (urban/rural). For those following the recommendations of the ACSM (at least four times per week) and those who did not practice that minimum of physical exercise (less than four times per week).

### Data Analysis

A descriptive statistic (mean, standard deviation, and frequency) was used and inferential statistics of the data were calculated. An analysis of variance of two factors (2 × 2), gender (male and female) and practice of physical activity according to the ACSM (a physical activity is frequently practiced according to the ACSM, or not). Bonferroni *post hoc* analysis was used. The level of significance was set in *p* < 0.05. The classification to measure the magnitude of the effect size was used (Ferguson, 2009): no effect (η^2^ < 0.04), minimum effect (0.04 < η^2^ < 0.25), moderate effect (0.25 < η^2^ < 0.64) and strong effect (η^2^ > 0.64). The statistical analysis was completed with SPSS software (version 21.0).

## Results

In Table 1, the means and standard deviations of aggressiveness in men and women who practiced the minimum frequency of physical exercise per week recommended by the ACSM (at least four times per week) and those who did not practice that minimum of physical activity (less than four times per week), are shown.

**TABLE 1:** Descriptive values of aggressiveness in women and men, according to the frequency of practice of physical activity.

**Gender**	**Practice PA ACSM**	***M***	***SD***	***N***
Women	Not practice PA. ACSM	6.47	13.60	353
	Practice PA. ACSM	8.44	14.37	281
	Total	7.34	13.97	634
Male	Not practice PA ACSM	5.03	11.08	344
	Practice PA ACSM	8.61	14.99	270
	Total	6.60	13.06	614
Total	Not practice PA ACSM	5.76	12.43	697
	Practice PA ACSM	8.52	14.66	551
	Total	6.98	13.53	1248

After the implementation of the analysis of variance of two factors (2 × 2), it was observed that the effect of the interaction between both variables was not significant (*F*_1,1244_ = 1.093, *p* = 0.296, η^2^= 0.001), and, therefore, our findings do not support that the interaction between gender and frequency of physical activity had an impact on the levels of aggressiveness in bullying.

From the perspective of the between-subjects factor gender, were not statistically significant differences between them (*F*_1,1244_ = 0.687, *p* = 0.407, η^2^ = 0.001). These data showed that women and men had similar values of aggressiveness. In this sense, there were not statistically significant differences between men and women who practiced PA according to the ACSM (*F*_1,1244_ = 0.021, *p* = 0.885, η^2^ = 0.000) nor between those who did not (*F*_1,1244_ = 1.989, *p* = 0.159, η^2^ = 0.002).

On the other hand, from the perspective of the between-subjects factor of practicing PA in accordance with the ACSM, the students who practiced PA according to the ACSM showed higher statistically significant values (*F*_1,1244_ = 13.083, *p* = 0.000, η^2^ = 0.051) with reference to those who did not practice PA according to the ACSM. These data showed that those students who practiced it have higher values of aggressiveness in comparison to those students who did not. In this sense, there were statistically significant differences in men between those who practiced PA according to the ACSM and those who did not (*F*_1,1244_ = 10.688, *p* = 0.001, η^2^ = 0.049), and a tendency toward significance in women (*F*_1,1244_ = 3.364, *p* = 0.067, η^2^ = 0.043). In both cases, there were higher values in men and women who practiced PA according to the ACSM than in those students who did not practice it.

**FIGURE 1:**
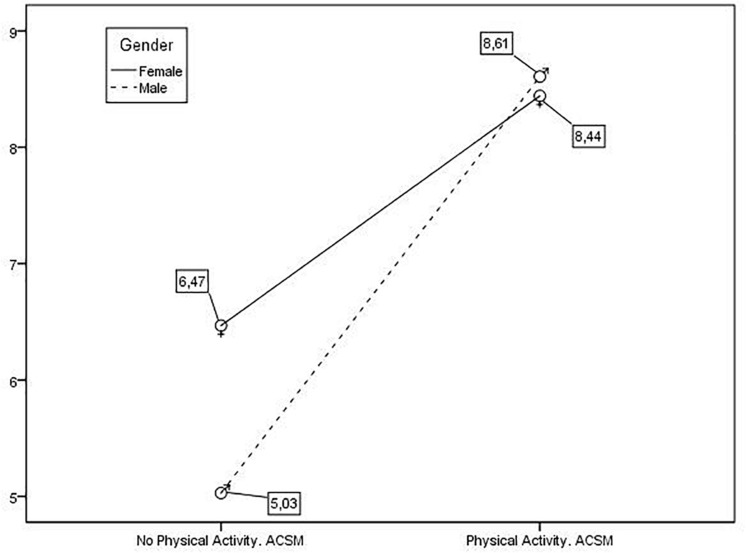
Values of Aggressiveness according to Gender/Practice of PA according to ACSM.

In Figure 1, it can be seen that amongst the students not practicing PA according to the ACMS, women tended to have higher values of aggressiveness, while men showed higher values when practicing PA according to the ACSM.

In Table 2, the means and standard deviations of victimization in men and women, who practiced physical activity at least four times per week (as recommended by the ACSM), and those who did not practice that minimum frequency, can be seen.

**TABLE 2:** Descriptive values of Victimization in women and men according to the frequency of practice of physical activity.

**Gender**	**Practice PA ACSM**	***M***	***SD***	***N***
Women	Not practice PA ACSM	4.88	11.95	353
	Practice PA ACSM	5.34	10.92	281
	Total	5.08	11,50	634
Male	Not practice PA ACSM	11.67	17.03	344
	Practice PA ACSM	11.05	16.38	270
	Total	11.40	16.73	614
Total	Not practice PA ACSM	8.23	15.06	697
	Practice PA ACSM	8.14	14.15	551
	Total	8.19	14.66	1248

After the implementation of the analysis of variance of two factors (2 × 2), gender and practice of physical activity according to ACSM, it was observed that the effect of the interaction between both variables was not significant (*F*_1,1244_ = 0.435, *p* = 0.510, η^2^ = 0.000), and, therefore, our findings do not support that the interaction between both factors had an impact on the levels of victimization in bullying.

From the perspective of the between-subjects factor gender, women had lower levels of victimization than men with statistically significant differences (*F*_1,1244_ = 58.531, *p* = 0.000, η^2^ = 0.055). These data showed that women had greatly inferior values with respect to men. In this sense, there were statistically significant differences either between men and women practicing PA according to the ACSM (*F*_1,1244_ = 21.875, *p* = 0.000, η^2^ = 0.047) and those not practicing PA according to the ACSM (*F*_1,1244_ = 39.109, *p* = 0.000, η^2^ = 0.060).

**FIGURE 2:**
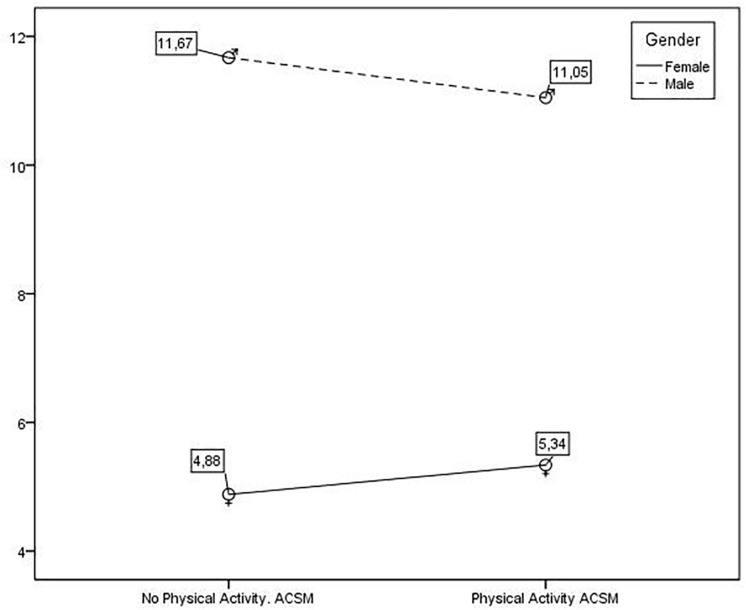
Values of Victimization according to Gender/Practice of PA according to ACSM.

On the other hand, from the perspective of the between-subjects factor of the between-subjects factor of practicing PA in accordance with the ACSM, the students who practiced PA according to the ACSM showed slightly inferior values which were not statistically significant (*F*_1,1244_ = 0.010, *p* = 0.920, η^2^ = 0.000) with reference to those who did not practice PA according to the ACSM. These data showed that those students who practiced it had very similar values to those students who did not. In this sense, there were not statistically significant differences between those who practiced PA according to the ACSM and those who did not practice it, neither in men (*F*_1,1244_ = 0.284, *p* = 0.594, η^2^ = 0.000) nor in women (*F*_1,1244_ = 0.159, *p* = 0.690, η^2^ = 0.000). In Figure 2, it is shown that women, either practicing or not PA according to the ACSM, had lower values of victimization than men.

## Discussion

Due to that there are scarce studies which relate bullying in the practice of PA, outside school, the aim of this study was to analyze the impact of the amount of healthy physical activity on the risks for the students directly involved in bullying (bully and victim) according to gender. So the data obtained follow the line of previous researches which evince that both men and women are involved in the different forms of aggression (Chacón-Cuberos et al., 2015; Martínez-Martínez et al., 2017; López-Castedo et al., 2018; Medina Cascales and Reverte Prieto, 2019). Although the data of the present study indicate that an effect of the interaction between aggressiveness and gender is not appreciated, the descriptive data (Figure 1) reflect trends that could be confirmed with more subjects, so that the practice of physical activity modifies the levels of aggressiveness more in men than in women. Those teenagers who practiced sport on a regular basis showed a moderate effect rate of overt aggressiveness than the sedentary adolescents (Zurita-Ortega et al., 2015), since in the competitive activities, students replicate violent situations (Subdirección General de Promoción Deportiva y Deporte Paralímpico, 2015). Moreover, the students who practice team sports (non-competitive) show a lesser risk of developing aggressive and deviant conducts (Pelegrín et al., 2010). In relation to victimization, significant differences by gender, in contrast with other studies, were found (Espelage et al., 2004; Rodkin and Berger, 2008). Women show lower levels of victimization, both among those who practice PA as among those who do not. In both genders, the practice of PA does not reduce the levels of victimization. However, it seems that the amount of physical activity carried out and the type of sport practiced can act as regulators in the victimization for bullying (Medina Cascales and Reverte Prieto, 2019). Indeed in our study, men tended to do more sport than women (Chahín-Pinzón and Libia, 2011; Hernando et al., 2013; Hutchens et al., 2016; Ramos et al., 2016; Martínez-Martínez et al., 2017; Medina et al., 2018).

In conclusion due to in adolescence, some lifestyles, which could put at risk the quality of life, may appear, therefore, the practice of a physical activity is a fundamental factor in health promotion in childhood and adolescence (Ramos et al., 2016). Most adolescents practice team sports (non-competitive) and this provides greater possibilities of enjoyment and, at the same time, promotes social relationships (González et al., 2016). According to Gómez (2007), it is necessary that the different professionals (psychologists, educators, etc.) tackle violence and the attitude toward it in the physical sporting context. Likewise, it is essential to promote an active lifestyle among teenagers, mainly among those from a low socioeconomic level and, particularly, among women (Ramos et al., 2016). Therefore, it is key to reinforce attitudes which favor healthy habits (Campo-Ternera et al., 2017; Lima-Serrano et al., 2018; Medeiros et al., 2018; Santos et al., 2018; Palomino-Devia et al., 2018) so antisocial and criminal behaviors can be minimized (Pelegrín et al., 2010; Gázquez et al., 2015). The practice of physical activity (non-competitive) entails the promotion of essential values for a peaceful coexistence and socialization, being an ideal tool to diminish the cases of violence (Martínez-Baena and Faus-Bosca, 2018; Medina Cascales and Reverte Prieto, 2019). Consequently, the role of PE teachers is noteworthy in the promotion of proactive strategies to prevent bullying through training sessions and competitions, which allow working in the resolution of conflicts that is to say avoiding competitive sports (Hand, 2016; López-Castedo et al., 2018) or even implementing in education centers programs which promote school coexistence, plans, protocols (Ortega-Ruiz and Córdoba-Alcaide, 2017), prosociality, empathy and emotional control (Gómez-Ortiz et al., 2017), programs on forgiveness in the prevention and treatment of bullying (Barcaccia et al., 2018), among others. Moreover, it is also necessary to consider that the prevention programs centered on students with a certain vulnerability (e.g., deficient gross motor skills) could diminish bullying (Bejerot et al., 2011, 2013; Healy, 2014).

On the whole, as a limitation of this research study can be pointed out the fact that the present study used a standard cross-sectional methodology even some of the data are self-reported these results may be biased due to distorted responses or social desirability. Finally, it would be desirable to use at the same time other assessment instruments which would allow identification of other variables as well as evaluation of the teaching staff and the family.

On the other hand, future research could consider other variables such as cyberaggression, parental control (Álvarez-García et al., 2018), self-concept of the physical condition and perceived competence, mental health (Palomino-Devia et al., 2018), physical activity carried out by parents (Loch et al., 2015; Castro-Sánchez et al., 2016; González et al., 2016; Greca et al., 2016), the impact of the socioeconomic inequalities in lifestyles and health (Moreno-Maldonado et al., 2016; Chzhen et al., 2018), resilience (Moreno et al., 2016), longitudinal studies, etc., Also, longitudinal studies using multiple informants (e.g., adolescence, peer, parent, coach, and teacher) are needed to establish true casual connections among variables.

## Ethics Statement

This study was carried out in accordance with the recommendations of the Oviedo Agreement by the Ethic Committee for Clinic Investigations of the University of Murcia and with a written informed consent from all participants. Parents also gave written informed consent in accordance with the Declaration of Helsinki.

## Author Contributions

IM contributed to the fieldwork, analyzed the theoretical development, and wrote the manuscript. CR-E analyzed the theoretical development and wrote the manuscript. EO contributed to the fieldwork, analyzed the methodological treatment, and wrote the manuscript.

## Conflict of Interest Statement

The authors declare that the research was conducted in the absence of any commercial or financial relationships that could be construed as a potential conflict of interest.
